# One year remission of type 1 diabetes mellitus in a patient treated with sitagliptin

**DOI:** 10.1530/EDM-14-0072

**Published:** 2014-09-01

**Authors:** Marcos M Lima-Martínez, Ernesto Guerra-Alcalá, Miguel Contreras, José Nastasi, Janelle A Noble, Constantin Polychronakos

**Affiliations:** 1 Departamento de Ciencias Fisiológicas, Universidad de Oriente, Ciudad Bolívar, Venezuela; 2 Unidad de Endocrinología, Diabetes, Metabolismo y Nutrición, Anexo A. Centro Médico Orinoco, Avenida Siegart, Ciudad Bolívar, 8001Venezuela; 3 Departamento de Medicina, Hospital Vargas, Caracas, Venezuela; 4 Centro Médico El Valle, Porlamar, Venezuela; 5 Servicio de Genética Médica, Universidad de Oriente, Ciudad Bolívar, Venezuela; 6 Children's Hospital Oakland Research Institute, Oakland, California, USA; 7 Departments of Paediatrics and Human Genetics, McGill University Health Centre, Montreal, Quebec, Canada

## Abstract

Type 1 diabetes mellitus (T1DM) is a chronic disease characterized by the autoimmune destruction of pancreatic β-cells. This paper describes the case of a 19-year-old male patient who presented with glutamic acid decarboxylase (GAD) antibody positive and diabetic ketoacidosis, which mandated intensive insulin treatment. Once the ketoacidosis was controlled, an oral dose of 100 mg of sitagliptin was administered once a day. Ketoacidosis was managed by insulin and insulin daily requirement began to dwindle after one month, until its complete withdrawal at 8 weeks, when partial remission was reached. The patient has now remained on sitagliptin treatment alone for a year, without requiring insulin. The benefit observed with this medication is possibly associated with its immunological effects. Inhibition of dipeptidyl peptidase 4 in animal models deregulates the Th1 immune response, increases secretion of Th2 cytokines, activates CD4+CD25+FoxP3+ regulatory T-cells, and prevents IL17 production.

**Learning points:**

- The use of insulin-dose-adjusted HbA1c constitutes the best way to define partial remission in T1DM patients.
- The use of sitagliptin in T1DM patients could help to decrease daily requirement of insulin by delaying β-cell loss and improving endogenous insulin production.
- The determination of antibodies against insulin, islet cells, and GAD permits differentiation of T1DM patients from those with atypical or ketosis-prone diabetes.

## Background

Type 1 diabetes mellitus (T1DM) is a chronic disease characterized by the autoimmune destruction of pancreatic β-cells in genetically susceptible subjects, which results in absolute insulin deficiency. This pathology is usually diagnosed between the age of 6 months and adulthood, and is clinically manifested through polyuria, polydipsia, and weight loss associated with glycosuria and ketonuria (1).

Several agents used to reestablish immunological tolerance over the past few years have successfully prevented and even reverted T1DM in nonobese diabetic mice; however, these outcomes have not been achieved in humans (1).

This paper describes the case of a male patient aged 19 who presented with T1DM and whose condition has been remitted for a year, being currently treated only with sitagliptin.

## Case presentation

The case is a 19-year-old male patient from Ciudad Bolívar, Venezuela, without any familial history of diabetes, presented with polyuria, polydipsia, and weight loss (16 kg) with 3 months of evolution.

The physical examination showed a weight of 61 kg; a height of 1.71 m; BMI of 20.8 kg/m^2^; a waist circumference of 76 cm; blood pressure at 100/60 mmHg.

## Investigation

The blood tests showed: fasting blood glucose: 432 mg/dl; HbA1c: 12.3%, basal insulin: 3.2 mUI/ml, C-peptide: 1.2 ng/ml, venous pH: 7.2, bicarbonate: 13 mEq/l, total cholesterol: 178 mg/dl, triglycerides: 196 mg/dl, HDL cholesterol: 41 mg/dl, and LDL cholesterol: 97 mg/dl. Urinalysis revealed glycosuria and ketonuria. Glutamic acid decarboxylase (GAD) antibody resulted positive (46 U/ml, reference range 1–5), but islet cell antibody and anti-insulin tests were negative.

Human leukocyte antigen (HLA) genotyping for DR and DQ-encoding loci was carried out by next generation sequencing on the Roche 454 GS Junior platform as previously described (2) and resulted in the following genotypes: DQA1*01:01:01, DQA1*05:01:01; DQB1*02:01:01, DQB1*05:01:01; DRB1*03:01:01, DRB1*10:01:01; and DRB3*02:02:01.

Based on established patterns of linkage disequilibrium for these loci, the genotypes can be assigned to the following haplotypes: DRB1*03:01:01-DRB3*02:02:01-DQA1*05:01:01-DQB1*02:01:01 (DR3) and DRB1*10:01:01-DQA1*01:01:01-DQB1*05:01:01 (DR10).

## Treatment

An intensive s.c. regimen of both insulin glargine and insulin glulisine was prescribed at a dose of 0.5 units/kg per 24 h, reaching an adequate metabolic control in 72 h, after which sitagliptin at a dose of 100 mg was initiated with a frequency of once a day.

## Outcome and follow-up

Upon completion of the first month of treatment, the patient started to show a significant reduction in daily insulin requirement, until its complete discontinuance eight weeks after diagnosis, when the patient entered remission and continued on sitagliptin alone, reaching fasting plasma glucose concentrations between 70 and 130 mg/dl and an HbA1c of 7.8%. The insulin-dose-adjusted HbA1c, defined as actual HbA1c (%)+(4×insulin dose (units/kg per 24 h)) (3), was 7.8 (value defining partial remission ≤9). By this time, the patient had gained 7 kg of weight. The GAD antibody levels were significantly decreased (8 U/ml). The levels of C-peptide plasma concentration remained the same.

After one year of treatment with only 100 mg of sitagliptin, the blood test report of the patient shows the following values: HbA1c, 5.8%; fasting plasma glucose, 108 mg/dl; basal insulin, 2.6 mIU/ml; fasting C-peptide, 1.0 ng/ml; 2 h, 75 g postprandial glucose, 152 mg/dl, and an insulin value of 25.7 mIU/ml with a C-peptide of 4.3 ng/ml.

Currently, after 15 months on sitagliptin treatment, the patient's last assessment showed the following report: HbA1c, 6.3%; fasting plasma glucose, 122 mg/dl; basal insulin, 2.4 mIU/ml; fasting C-peptide, 0.9 ng/ml; 2 h, 75 g postprandial glucose 164 mg/dl, and an insulin value of 18.3 mIU/ml with a C-peptide of 3.9 ng/ml. The GAD-antibodies remain positive (6 U/ml).

## Discussion

The pathogenesis of T1DM is predicated on both genetic predisposition factors (predominantly HLA alleles) and the presence of autoantibodies in the serum against islet cells, insulin, tyrosine phosphatase (IA-2), and GAD (1). The subject was diagnosed with T1DM based on HLA alleles, the HbA1c test, and the presence of positive antiGAD. The haplotypes DRB1*03:01-DQA1*05:01 and DQB1*02:01 (DR3) are well established to be predisposing for T1DM, with odds ratios in the range of 3–5, depending on the population (4). A recent report has shown that DR3 haplotypes carrying the allele DRB3*02:02 are more predisposing than those carrying the allele DRB3*01:01 (2). Thus, the DR3 haplotype in the patient is consistent with T1DM autoimmunity and the presence of anti-GAD antibodies.

At the disease onset, between 10 and 70% of T1DM patients present a typical profile of diabetic ketoacidosis similar to that found in our patient upon hospitalization: blood glucose >200 mg/dl, venous pH <7.3, bicarbonate level <15 mEq/l, and ketonuria (1). The majority of ketoacids, especially acetoacetate and β-hydroxybutyrate, originate from the β-oxidation of fatty acids in the liver. T1DM patients have a marked deficiency of insulin and thus peripheral lipolysis and ketogenesis cannot be supressed.

The treatment of this disease aims at diminishing the acute and chronic complications associated with diabetes, and improving the quality of life of the patients. The Diabetes Control and Complications Trial (DCCT) demonstrated that early use of an intensive treatment with insulin can prolong β-cell reserve in affected individuals (5). Furthermore, preserving some β-cell residual function, measured through C-peptide secretion, allows for a decline in exogenous insulin requirements, thus reaching a honeymoon phase; however, recent evidence has demonstrated that the presence of diabetic ketoacidosis at T1DM onset is associated with a worse long-term metabolic regulation and a lower β-cell residual function despite the use of insulin (6), in sharp contrast with what has been observed in this patient, suggesting, perhaps, the influence of an additional factor on the patient's honeymoon period besides early and intensive use of insulin.

The definition of partial remission (honeymoon) in T1DM varies. It has been defined as requiring <0.5 units/kg per 24 h or an HbA1c ≤7.5%; however, each parameter on its own has the inconvenience that any change in treatment would easily influence patient classification. Therefore, it is easier to use the insulin-dose-adjusted HbA1c as definition of partial remission, as it comprehensively takes into account the metabolic control of the patient, the insulin dose, and its relation with β-cell function, this being more convenient than conventional definitions of partial remission (3). Regardless of the definition, the above-referenced patient has been in remission for 15 months without requiring exogenous insulin.

Sitagliptin was prescribed for this patient because a growing body of evidence has demonstrated that inhibitors of dipeptidyl peptidase 4 (DPP4) can diminish daily insulin requirements and improve metabolic control without exacerbating the risk of hypoglycemia in T1DM patients (7) (8). However, as far as we are concerned, there has been no publication of another case of remission without insulin in a T1DM patient treated with this drug.

Although it is perhaps much too early to conclusively assert that the unusual remission observed in just one case must be ascribed to sitagliptin, the findings in animal models suggest that the benefits observed in clinical trials of sitagliptin including T1DM patients are associated with the drug's immunologic effects. The lymphocytes have a membrane-associated protein with DPP4 activity, CD26, which has a marked influence on the development, migration, and production of cytokines by T-cells. Inhibition of DPP4 in animal models deregulates the Th1 immune response, increases secretion of Th2 cytokines, activates CD4+CD25+FoxP3+ regulatory T-cells, and prevents IL17 production (9).

**Figure 1:**
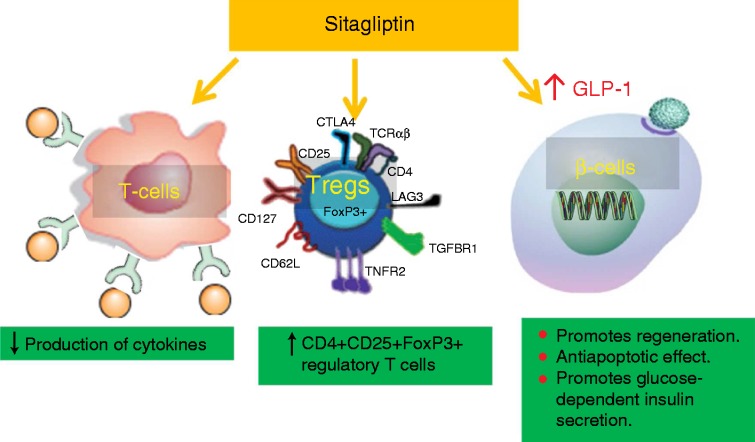
Pleiotropic effects of sitagliptin. In animal models, DPP4 inhibition by sitagliptin lowers the production of inflammatory cytokines by T-cells, increases CD4+CD25+FoxP3+ regulatory T-cells (Tregs) production and, by increasing GLP1 half-life promotes β-cell regeneration, inhibits apoptosis, and fosters glucose-dependent insulin secretion.

The immunoregulatory effects of DPP4 inhibition have been less-widely reported in humans; of late, however, the effects of sitagliptin in patients with psoriasis have been documented (10) (11). Interestingly, a case report showed that sitagliptin treatment on an autoimmune diabetic patient affected with stiff person syndrome lowered HbA1c significantly and decreased GAD antibody levels by ∼85% after 18 months (12). Besides, sitagliptin treatment increases the half-life of GLP1, which promotes glucose-dependent insulin secretion; inhibits α-cell glucagon release; and exerts an antiapoptotic effect on β-cells (Fig. 1). This patient currently presents lower basal plasma concentrations of insulin and C-peptide than the levels present at the time of his admission into the hospital, possibly owing to a slow but progressive autoimmune β-cell destruction. He maintains, however, a good response of both insulin and C-peptide upon stimulation with 75 g of glucose, which might be related to the dual effect of GLP1 on islet cells.

In 1987, Winter *et al*. (13) described a group of individuals, many of them of African or Latin American descent, who displayed spontaneous episodes of diabetic ketoacidosis and then reached normal ranges of glycemia and an improved β-cell insulin secretory capacity, thereby allowing oral hypoglycemic therapy. This form of diabetes was called atypical diabetes, or A^−^ B^+^ ketosis-prone diabetes. Similarities notwithstanding, the aforesaid patient does not have this kind of diabetes, since subjects with A^−^ B^+^ ketosis-prone diabetes lack the evidence of pancreatic autoimmunity, and most of them have a tendency to obesity with a familial history of diabetes mellitus.

## Patient consent

Written informed consent was obtained from the patient for publication of this case report.

## Author contribution statement

The following were the contributions made by the authors: M M Lima-Martínez, E Guerra-Alcalá, and M Contreras were involved in drafting of the manuscript; J Nastasi and J A Noble performed the genetic analysis; J A Noble and C Polychronakos were involved in critical revision of the manuscript; and M M Lima-Martínez was the patient's physician.
